# Comparison of two different minimally invasive techniques used in bladder stone surgery for preschool-aged children

**DOI:** 10.55730/1300-0144.5433

**Published:** 2022-08-04

**Authors:** İsmail YAĞMUR, Mehmet DEMİR, Bülent KATI, Eyyüp Sabri PELİT, Eser ÖRDEK, Halil ÇİFTÇİ

**Affiliations:** Department of Urology, Faculty of Medicine, Harran University, Şanlıurfa, Turkey

**Keywords:** Bladder stone, children, preschool-age, transurethral cystolithotripsy, percutaneous cystolithotomy, mini-percutaneous

## Abstract

**Background/aim:**

This study was designed to compare the outcomes of mini-percutaneous cystolithotomy (mPCL) and transurethral cystolithotripsy (TUCL) in treating bladder stones in preschool-aged children (≤6 years old).

**Materials and methods:**

Twenty-four patients treated with mPCL and 28 patients treated with TUCL for bladder stones were compared retrospectively. The operative and postoperative outcomes of both groups were analyzed.

**Results:**

The mean age and gender distribution were similar between the groups. The mean stone size was 16.5 ± 0.5 mm for the mPCL group and 14.9 ± 5.7 mm for the TUCL group (p = 0.318). The mean operative time was 41.1 ± 9.9 min for the mPCL group and 39.0 ± 12.3 min for the TUCL group (p = 0.182). Catheterization times and hospitalization times were statistically significantly longer in the mPCL group (p = 0.000). The rate of urinary retention after urethral catheter removal was significantly higher in the TUCL group (p < 0.05). Reintervention was performed for one patient in Group 1 due to urinary leakage and for five patients in Group 2 due to urinary retention. The stone-free rate (SFR) after a single procedure was 100% in the mPCL group and 89.3% in the TUCL group (p = 0.099). After auxiliary procedures performed for three patients, the overall SFR also reached 100% for the TUCL group.

**Conclusion:**

Both mPCL and TUCL are effective methods in the treatment of bladder stones of <30 mm in the preschool age group. Although TUCL has some advantages over mPCL, such as shorter hospital stays and catheterization times, there is a risk of urinary retention with increased stone sizes. It may be more advantageous to apply mPCL for the reduction of complications and reintervention rates, especially in small children with bladder stones of >20 mm.

## 1. Introduction

Bladder stones account for 5% of urinary system stones. Although pediatric bladder stones are rare in developed countries, they are still common in developing countries [1]. Genetic, metabolic, and environmental factors play a role in the etiology [2]. Bladders stones are more common in boys than girls, and anatomical factors such as urethral length and diameter are held responsible for this situation [3].

Open cystolithotomy (OC), shock wave lithotripsy (SWL), percutaneous cystolithotomy (PCL)/cystolithotripsy (PCCL), and transurethral cystolithotripsy (TUCL) methods are used in the treatment of pediatric bladder stones [4,5]. Each method has its own advantages and disadvantages [6]. Open surgery has disadvantages such as prolonged hospital stay, cosmetic deterioration due to suprapubic scarring, prolonged catheterization, need for analgesics, and risk of wound infection [7]. For these reasons, minimally invasive techniques have been widely adopted in the management of bladder stones to reduce the risk of complications and shorten hospital stay and recovery time [4]. The use of SWL, which is a minimally invasive method, is limited in children because it has disadvantages such as incomplete fragmentation of the stone, difficulty in the spontaneous passage, and the need for additional sessions and intervention [8]. Percutaneous techniques (i.e. PCL/PCCL), on the other hand, are a safe alternative with low morbidity and complication rates for overburdened bladder stones [7–9]. With advances in miniature endoscopes and intracorporeal lithotripters, however, there has been a gradual shift to less invasive transurethral procedures in the treatment of pediatric bladder stones [10].

We have been performing mini-percutaneous cystolithotomy (mPCL) using mini-nephroscopes and bladder trocars for about 10 years and we have previously reported on this technique [11]. For the last 5 years, we have been performing TUCL using compact cystoscopes and Ho-YAG lasers. The present study was designed to compare the results of these two minimally invasive methods in preschool children.

## 2. Materials and methods

This retrospective study was approved by the local ethics committee and was conducted in accordance with the Declaration of Helsinki (date and approval no: 21.12.2020, HRU/20.22.09). Computer records and patient files between January 2010 and December 2020 were reviewed retrospectively. In our clinic, we performed mPCL for all patients with bladder stones smaller than 30 mm between 2010 and 2016. After 2017, we performed TUCL for these patients. Children younger than 6 years of age who underwent mPCL or TUCL due to bladder stones smaller than 30 mm were included in the study. Patients with a history of urethral stricture, posterior urethral valve (PUV), neuropathic bladder, lower urinary system surgery, and/or follow-up of less than 6 months were excluded from the study.

The diagnosis of bladder stone was made by plain abdominal X-ray and ultrasonography (US). Computed tomography was performed in cases of clinical suspicion. Stone size was taken as the largest diameter on X-ray or US. Complete blood count, kidney function tests, urinalysis, and culture-antibiogram were performed for all patients.

The patients were divided into two groups. Group 1 consisted of patients for whom we applied mPCL and Group 2 consisted of patients who underwent TUCL. Age, gender, symptoms, blood results, urinalysis, stone size, operation time, durations of catheterization and hospital stay, and complications were recorded and the groups were compared in terms of these parameters. In addition, patient groups with and without reintervention were compared in terms of the above-mentioned parameters.

### 2.1. Surgical techniques

All procedures were performed by two experienced urologists (İY, HÇ) under general anesthesia and in the lithotomy position. All procedures were begun with urethrocystoscopy. This procedure provides direct endoscopic vision for percutaneous access and aids in the diagnosis of urethral stricture, PUV, or other lower urinary tract pathologies [10].

#### 2.1.1. Mini-percutaneous cystolithotomy

For mPCL, we applied our own techniques previously reported [11]. Bladder stones were detected in all patients at baseline by cystoscopy. The bladder was then filled to its maximum capacity with normal saline. Under cystoscopic control, the bladder was entered in a single step 2 cm above the pubic bone with an 18F dilator trocar and access sheath (Karl Storz GmbH & Co. KG, Tuttlingen, Germany). We did not use any dilatation technique or guide such as fluoroscopy and/or US. After the trocar was placed, its obturator was removed. A 12F nephroscope or 17F nephroscope (without sheath) (Karl Storz GmbH & Co. KG, Tuttlingen, Germany) was inserted through the trocar. After visualization of the bladder, the stones were fragmented with a pneumatic lithotripter (Vibrolith, Elmed, İstanbul, Turkey). Stone fragments were removed by holding them with forceps. At the end of the operation, a urethral catheter (6/8F) was placed and the suprapubic skin was closed with a primary suture.

#### 2.1.2. Transurethral cystolithotripsy

Cystoscopy was performed under general anesthesia and in the lithotomy position using an 8F 6° compact cystoscope (Karl Storz^®^, Tuttlingen, Germany). After visualizing the bladder stones, the stones were fragmented using a 30-W Sphinx Jr. Ho-YAG LISA laser (OmniGuide, Lexington, MA, USA) with 550-μm laser fiber. Stone removal was not performed. A urethral catheter (6/8F) was placed at the end of the procedure.

### 2.2. Follow-up

In the percutaneous method, the urethral catheter was removed on the second day after the operation, and in the transurethral method, on the first day after the operation. Stones were sent for analysis. Parents were asked to bring their children to our outpatient clinic for follow-up in the second week and again 6 months after the operation. “Stone-free” was defined as the absence of stones on plain abdominal X-ray and US. Uroflowmetry was performed according to indications for patients whose voiding training was completed.

### 2.3. Statistical analyses

Values of mean, standard deviation, median, minimum, maximum, frequency, and percentage were used for descriptive statistics. The distribution of variables was checked with Kolmogorov-Smirnov testing. Independent samples t-tests and Mann-Whitney U tests were used for the comparison of quantitative data. Chi-square tests were used for the comparison of qualitative data. SPSS 27.0 was used for statistical analyses. The statistical significance level was taken as p < 0.05.

## 3. Results

A total of 52 patients (48 boys and 4 girls) were included in the study. Demographic data and operation results of the patients are summarized in Table 1. The mean age of the patients was 32.0 ± 19.4 months and mean stone size was 15.6 ± 5.6 mm. The most common presenting symptoms were urinary retention, hematuria, abdominal pain, and restlessness (Table 1). Preoperative urinary infection was detected in five cases. The isolated microorganisms were *Escherichia coli* (n = 3), *Pseudomonas aeruginosa* (n = 1), and *Klebsiella pneumoniae* (n = 1). These cases were treated according to the culture-antibiogram results and the urine was sterilized before the operations.

Group 1 (mPCL) included 24 patients while Group 2 (TUCL) included 28 patients. There was no statistically significant difference between the two groups in terms of age, gender distribution, stone size/number, or operation time (p > 0.05) (Table 1). The durations of both urethral catheterization and hospital stay were significantly longer in Group 1 than Group 2 (p < 0.05). The rate of urinary retention after urethral catheter removal was significantly higher in the TUCL group than in the mPCL group (p < 0.05). There was no statistically significant difference between the two groups in terms of other complications (p > 0.05) (Table 1).

Reintervention was performed for one patient in Group 1 due to urinary leakage and five patients in Group 2 due to urinary retention (Table 1). Recatheterization was performed for three of these patients, cystoscopy and bladder washing for two, and mPCL with cystoscopy for one. Values for mean stone size, operation time, and hospital stay were significantly higher in the group with reintervention (n = 6) than the group without reintervention (p < 0.05) (Table 2). However, the difference between the two groups in terms of reintervention rates was not statistically significant (p > 0.05).

Mean follow-up time was 19.0 ± 12.4 months. Stone-free status was achieved for all patients and no obstructive uroflowmetry findings were observed in any cases. In stone analyses, the main components were ammonium acid urate 50% (12/24), calcium oxalate 21% (5/24), and struvite 17% (4/24).

## 4. Discussion

The most commonly used minimally invasive methods in the treatment of pediatric bladder stones are TUCL and PCL/PCCL [4,12]. However, due to the narrow diameter of the urethra in young children, it is thought that the transurethral approach may be risky [7]. Therefore, the PCL/PCCL method, which has less morbidity compared to open surgery and fewer restrictions than the transurethral endoscopic method, still maintains its popularity [4]. However, there are few studies in the literature comparing these two methods, especially in young children [5,9].

Percutaneous bladder stone surgery is an effective and safe method with high stone-free, low morbidity, and low complication rates [8]. However, there is no standardized technique in the literature. With the aim of accessing the bladder, Hassan et al. used a 30F Amplatz sheath, Dhabalia et al. used a 21F trocar, Mishra et al. used a 15F access sheath, and Bodakci et al. used a 14-gauge angiocath needle [2,9,13,14]. Gan et al. reported that they used fluoroscopy in the dilatation phase in their minimally invasive PCL series of 15 patients, all of whom were younger than 1-year-old and male [15]. It is understood that not only variables related to the patient and the stone but also variables of surgical experience, the center’s available equipment, and the cost are effective in the selection of the treatment method. Our percutaneous technique has advantages such as providing safe access to the bladder without any guide or dilation, low risk of iatrogenic urethral stricture, no ionizing radiation, and no need for iodinated contrast material [11]. In addition, we evaluate urethra and bladder pathologies as we perform cystoscopy. In our own series, with this procedure, we found that two of the patients had urethral stricture, one had PUV, and one had a stone in the ureterocele. We treated those additional pathologies and excluded these patients from the study, ensuring that only patients with primary bladder stones were included.

When the literature is reviewed, it is seen that there is no standardized technique for TUCL. Aboulela et al. reported that they fragmented stones with an 11F cystoscope and laser and washed the bladder with the help of a sheath [6]. Mishra et al. reported that they performed lithotripsy using 4.5/6F cystoscopes or 6/7.5F ureteroscopes and holmium-YAG lasers [9]. Isen et al. reported that they used 8/9.8F or 9.5F ureteroscopes and pneumatic lithotripters [16]. We used an 8F 6° compact cystoscope (Karl Storz^®^, Tuttlingen, Germany) and Ho-YAG laser in our study. We think that this instrument is more ergonomic for both the patient and the operator since the diameter of the instrument is designed for the pediatric urethra and the working channel is straight, making fiber use easier, while the instrument’s length is shorter than that of ureteroscopes.

The mean operation time was found to be similar between the groups (p < 0.05). In addition, these mean times are similar to those reported in other studies in the literature [7,9,12,17]. Since we removed the urethral catheters later in Group 1, the durations of both catheterization time and hospital stay were higher than those in Group 2 and the differences were statistically significant (p < 0.05). Salah et al. reported longer mean catheterization times and mean hospitalization times (2 days and 2.7 days, respectively) in their series of 155 cases of pediatric PCL results, similar to our study [8]. Pişkin et al., in their study comparing PCCL and TUCL results in prepubertal children, reported that the mean hospitalization time was slightly longer in the PCCL group but that the difference was not statistically significant [12]. In contrast to these studies, Mishra et al. removed the Foley catheter at the postoperative 24th h in both their mini-PCCL and TUCL groups and discharged the patients the following day. Thus, they showed that hospitalization times could be reduced for patients treated by mini-PCCL, similarly to those of TUCL [9]. In light of this study and based on the encouraging results, we hope to remove the Foley catheters earlier in our future cases and thus shorten the hospital stay.

All patients in the current study had complete stone-free status at the 2nd and 6th month postoperatively. The stone-free rate (SFR) in Group 1 (mPCL) was 100% in the early postoperative period, as the fragments were easily removed from the suprapubic route with the help of forceps. However, three of the patients in the TUCL group who developed urinary retention following postoperative catheter removal required repeated cystoscopy. In two of these cases, only bladder washing was performed due to the presence of clinically insignificant stone fragments, and mPCL was performed for one patient due to the presence of a large stone fragment in the bladder. This stone suggested a residual fragment remaining after the first TUCL procedure. However, due to the round and regular shape of the stone, it also suggested the possibility of a radiolucent ureteral stone that could not have been detected during the initial diagnostic evaluation and spontaneously fell into the bladder. With these three cases, the SFR without reintervention in Group 2 was calculated as 89.3%, even if stone-free status was not achieved after the first procedure. The difference between the groups was not statistically significant (p = 0.099). Mishra et al. compared the results of mini-PCCL (n = 16) and TUCL (n = 15) in preschool children and reported an early SFR of 86.6%, similar to the rate obtained in our study [9].

Complications such as urinary leakage, intraperitoneal bladder perforation, bowel perforation, and paralytic ileus have been reported after the application of percutaneous methods [7,8,18]. In our series, recatheterization was performed due to urinary leakage (Clavien complication rate 1b) in only one case in the mPCL group (n = 24). Al-Marhoon et al. also reported that 1/27 patients had urinary leakage in their series [7]. Spontaneous recovery was observed after a 5-day Foley insertion.

In studies using the transurethral approach, early complications such as bladder perforation, urinary retention, and conversion to the percutaneous method and late complications such as urethral stricture have been reported [1,7,9,12]. Mishra et al. reported that they converted to mPCCL intraoperatively in one patient in the TUCL group due to the size of the fragments, and they performed cystoscopy again in another patient due to the presence of residual fragments during follow-up. The Clavien 3b complication rate in that study was found to be 2/15 (13.3%). In our study, two patients in the TUCL group (n = 28) underwent repeated cystoscopy and bladder irrigation, and one patient underwent mPCL due to large fragments. The Clavien 3b complication rate was 3/28 (10.7%), similar to the rate obtained in the series of Mishra et al. [9].

Although the mean stone size in their TUCL series was smaller than that in our series (12.1 ± 2.4 mm vs. 14.9 ± 5.7 mm), Pişkin et al. reported that 3/30 (10%) patients developed postoperative recurrent urinary retention. All three of those patients were under the age of 3 years and the mean stone size was 12 mm. They attributed those cases of urinary retention to dysuria caused by cystoscopy or spontaneous passage [12]. In other studies, with patients of older age groups, less or no urinary retention was reported. Khosa et al. found a urinary retention rate of 1% in their study, which included pediatric patients up to the age of 15 years [19]. Yıldız et al., on the other hand, did not report any postoperative urinary retention in a large series of 401 patients, including only adults, for whom they applied three different endoscopic treatments [20].

In our series, postoperative urinary retention developed in 5/28 (17.8%) patients in the TUCL group. While the median age of these patients was 35 months, the median stone size was 23 mm (range:20–25 mm) and the stones were larger than 20 mm in all cases. For 3/5 of these patients, bladder washing (n = 2) or mPCL (n = 1) was performed together with cystoscopy, since a Foley catheter could not be inserted. The other 2/5 patients were treated conservatively with only recatheterization. The mean values of stone size and operative time for these patients were significantly higher than those observed in the nonreintervention group (p < 0.05). We think that this is related to the fact that patients with urinary retention have lower mean age and larger stone size. In addition, it can be said that urethral edema and dysuria develop due to prolonged operation time. Therefore, we suggest that stone size, age, small urethral calibration, and voiding function are all important factors in the development of postoperative urinary retention. No late complications such as urethral stricture were observed in any of the cases in our series.

This study has some limitations. It is a retrospective study, there was no randomization, the surgeries were not all performed by a single surgeon, and stone analysis could not be performed for all patients. However, its strengths are that the number of cases is considerably large compared to previous works in the literature, it includes a younger age group, it is a comparative study, and standard techniques were used in both the mPCL and TUCL groups.

In conclusion, both of these minimally invasive methods are effective and safe in the treatment of bladder stones in preschool children. TUCL appears to be advantageous in terms of short catheterization and short hospitalization durations. However, it should be kept in mind that urinary retention may develop in cases of stones larger than 20 mm and mPCL should be preferred primarily in order to prevent that complication. There is a need for multicenter, large patient series and prospective studies to determine cut-off values for stone size in the selection of surgical methods, especially for children in younger age groups.

## Figures and Tables

**Table 1:** Demographic data and operation results of the groups.

		Group 1 (mPCL)				Group 2 (TUCL)				P
		Mean ± SD	n	%	Median	Mean ± SD/	n	%	Median	
Age (months)		30.5 ± 16.9			31.0	33.3 ± 21.5			25.5	0.594 t
Gender	Female		1	4.2%			3	10.7%		0.615 ^x2^
	Male		23	95.8%			25	89.3%		
Stone size (mm)		16.5 ± 5.5			15.0	14.9 ± 5.7			15.0	0.318 m
Operative time (min)		41.1 ± 9.9			37.5	39.0 ± 12.3			35.0	0.182 m
Catheterization time (days)		2.6 ± 1.2			2.0	1.5 ± 1.3			1.0	**0.000** m
Hospital stay (days)		3.5 ± 1.9			3.0	2.0 ± 2.2			1.0	**0.000** m
**Symptoms**										
Urinary retention			7	29.2%			5	17.9%		0.335 ^x2^
Hematuria			5	20.8%			6	21.4%		0.958 ^x2^
Discomfort			5	20.8%			6	21.4%		0.958 ^x2^
Fever			2	8.3%			4	14.3%		0.503 ^x2^
Abdominal pain			1	4.2%			4	14.3%		0.217 ^x2^
Urinary infection			2	8.3%			2	7.1%		1.000 ^x2^
Dysuria			1	4.2%			1	3.6%		1.000 ^x2^
Crying			0	0.0%			2	7.1%		0.493 ^x2^
Incidentally discovered			1	4.2%			0	0.0%		0.462 ^x2^
Vomiting			0	0.0%			1	3.6%		1.000 ^x2^
**Postoperative complications**										
Hematuria			5	62.5%			3	33.3%		0.347 ^x2^
Fever			3	37.5%			3	33.3%		0.858 ^x2^
Urinary retention			0	0.0%			5	55.6%		**0.029** ^x2^
Urinary infection			1	12.5%			3	33.3%		0.576 ^x2^
Urine leakage			1	12.5%			0	0.0%		0.471 ^x2^
Reintervention	−		23	95.8%			23	82.1%		0.123 ^x2^
	+		1	4.2%			5	17.9%		
Initial stone-free rate				100%				89.3%		0.099 ^x2^
Complete stone-free rate				100%				100%		

Statistically significant results are presented with bold italics (p < 0.05).

T Independent samples t-test;

m Mann-Whitney U test;

X2 chi-square (Fisher test).

**Table 2:** Demographic data and operation results of patients with and without reintervention.

		Re-intervention (−)				Re-intervention (+)				P
		Mean ± SD	n	%	Median	Mean ± SD	n	%	Median	
Age (months)		31.0 ± 19.8			25.5	39.5 ± 15.8			35.5	0.319 t
Gender	Female		4	8.7%			0	0.0%		1.000 ^x2^
	Male		42	91.3%			6	100.0%		
Stone size (mm)		14.9 ± 5.3			15.0	20.8 ± 5.6			23.3	**0.017** m
Operative time (min)		38.5 ± 10.4			35.5	51.2 ± 10.9			55.0	**0.015** m
Catheterization time (days)		1.9 ± 1.1			2.0	2.7 ± 2.7			1.0	0.770 m
Hospital stay (days)		2.3 ± 1.6			2.0	5.8 ± 3.7			5.0	**0.006** m
Cystoscopy			0	0.0%			2	33.3%		
Recatheterization			0	0.0%			3	50.0%		
mPCL			0	0.0%			1	16.7%		

Statistically significant results are presented with bold italics (p < 0.05).

T Independent samples t-test;

m Mann-Whitney U test;

X2 chi-square (Fisher test).

mPCL: Mini-percutaneous cystolithotomy.

## Abbreviations

PCL: Percutaneous cystolithotomy
mPCL: Mini-percutaneous cystolithotomy
PCCL: Percutaneous cystolithotripsy
mPCCL: Mini-percutaneous cystolithotripsy
TUCL: Transurethral cystolithotripsy
SWL: Shockwave lithotripsy
